# Gene Expression Landscape of SDH-Deficient Gastrointestinal Stromal Tumors

**DOI:** 10.3390/jcm10051057

**Published:** 2021-03-04

**Authors:** Valentina Indio, Angela Schipani, Margherita Nannini, Milena Urbini, Alessandro Rizzo, Antonio De Leo, Annalisa Altimari, Valerio Di Scioscio, Daria Messelodi, Giuseppe Tarantino, Annalisa Astolfi, Maria Abbondanza Pantaleo

**Affiliations:** 1 “Giorgio Prodi” Cancer Research Center, University of Bologna, 40138 Bologna, Italy; valentina.indio2@unibo.it (V.I.); giuseppetara91@gmail.com (G.T.); 2 Department of Experimental, Diagnostic and Specialty Medicine, University of Bologna, 40138 Bologna, Italy; angela.schipani2@unibo.it (A.S.); alessandro.rizzo11@studio.unibo.it (A.R.); 3 Division of Oncology, IRCCS—Azienda Ospedaliero Universitaria di Bologna, 40138 Bologna, Italy; margherita.nannini@aosp.bo.it (M.N.); maria.pantaleo@unibo.it (M.A.P.); 4 Biosciences Laboratory, IRCCS Istituto Romagnolo per lo Studio dei Tumori (IRST) “Dino Amadori”, 47014 Meldola, Italy; milena.urbini@irst.emr.it; 5 Pathology Unit, IRCCS—Azienda Ospedaliero Universitaria di Bologna, 40138 Bologna, Italy; antonio.deleo@unibo.it; 6 Laboratory of Oncologic Molecular Pathology, IRCCS—Azienda Ospedaliero Universitaria di Bologna, 40138 Bologna, Italy; annalisa.altimari@aosp.bo.it; 7 Radiology Unit, IRCCS—Azienda Ospedaliero Universitaria di Bologna, 40138 Bologna, Italy; valerio.discioscio@aosp.bo.it; 8 Department of Medical and Surgical Sciences, S. Orsola-Malpighi Hospital, University of Bologna, 40138 Bologna, Italy; daria.messelodi2@unibo.it; 9 Department of Translational Medicine, University of Ferrara, 44121 Ferrara, Italy

**Keywords:** SDH-deficient, gastrointestinal stromal tumor, GIST, fibroblast growth factor receptor, hypoxia, pheochromocytoma/paraganglioma, immune profile

## Abstract

Background: About 20–40% of gastrointestinal stromal tumors (GISTs) lacking KIT/PDGFRA mutations show defects in succinate dehydrogenase (SDH) complex. This study uncovers the gene expression profile (GEP) of SDH-deficient GIST in order to identify new signaling pathways or molecular events actionable for a tailored therapy. Methods: We analyzed 36 GIST tumor samples, either from formalin-fixed, paraffin-embedded by microarray or from fresh frozen tissue by RNA-seq, retrospectively collected among KIT-mutant and SDH-deficient GISTs. Pathway analysis was performed to highlight enriched and depleted transcriptional signatures. Tumor microenvironment and immune profile were also evaluated. Results: SDH-deficient GISTs showed a distinct GEP with respect to KIT-mutant GISTs. In particular, SDH-deficient GISTs were characterized by an increased expression of neural markers and by the activation of fibroblast growth factor receptor signaling and several biological pathways related to invasion and tumor progression. Among them, hypoxia and epithelial-to-mesenchymal transition emerged as features shared with SDH-deficient pheochromocytoma/paraganglioma. In addition, the study of immune landscape revealed the depletion of tumor microenvironment and inflammation gene signatures. Conclusions: This study provides an update of GEP in SDH-deficient GISTs, highlighting differences and similarities compared to KIT-mutant GISTs and to other neoplasm carrying the SDH loss of function. Our findings add a piece of knowledge in SDH-deficient GISTs, shedding light on their putative histology and on the dysregulated biological processes as targets of new therapeutic strategies.

## 1. Introduction

Succinate dehydrogenase deficient (SDH-deficient) gastrointestinal stromal tumors (GISTs), as defined by the expression loss of the subunit B of the succinate dehydrogenase complex, account for approximately 20% to 40% of all *KIT/PDGFRA* wild-type (WT) GISTs and 5% of all GISTs [1]. The SDH deficiency is mainly due to mutations in one of the four SDH mitochondrial complex subunits, *SDHA, SDHB, SDHC,* and *SDHD* [1,2,3]. Most SDHx mutations in GIST disease are germline, in particular, germline mutations in *SDHB, SDHC,* and *SDHD* occur in about 20–30% of SDH-deficient GISTs and may be referred to as Carney–Stratakis syndrome [2]. Rarely, an epigenetic mechanism may occur, such as the recurrent aberrant DNA methylation of *SDHC* seen in GISTs associated to the Carney triad, which is a rare condition characterized by synchronous or metachronous occurrence of GISTs, paragangliomas, and pulmonary chondromas [4,5,6]. Several studies reported that SDH-deficient GISTs are exclusively located in the stomach with mainly multifocal primary localization, frequently present lymph node involvement, and generally affect the younger population. Moreover, SDH-deficient GISTs present an indolent course even when multiple metastases are present [7].

Currently, data on the molecular background of the SDH-deficient GIST shows that this disease may be considered as a unique entity among the GISTs [8]. No specific medical therapy is available in this subset when SDH-deficient GISTs are recurrent or metastatic, but the standard flow chart of GIST treatment is still recommended even though antiangiogenic drugs seem to be the most effective in terms of control of the disease.

The aim of this work is to uncover the gene expression profile of the SDH-deficient GIST subtended to tumor development and invasion in order to identify new signaling pathways or molecular events actionable for a tailored therapy.

## 2. Materials and Methods

Thirty-six GIST tumor samples, from formalin-fixed, paraffin-embedded (FFPE) or fresh frozen tissue, were retrospectively collected and analyzed.

Fresh frozen tissue specimens (25 samples) were collected during the surgical operation, snap-frozen in liquid nitrogen, and stored at −80 °C until RNA extraction. Formalin-fixed, paraffin-embedded tissue blocks (13 samples) were obtained by fixing the surgical specimens in 10% NBF (formalin solution, neutral buffered) for no less than 6 h, then dehydrated and included in paraffin. Expert pathologists reviewed all samples, and the molecular alteration was detected by the routine GIST diagnostic panel by Sanger sequencing. Moreover, the SDH-mutant samples were tested by immunohistochemistry (IHC) in order to prove the negativity of SDHB staining.

The cohort consists of two distinct molecular subgroups of GISTs: KIT-mutant (29 cases) and SDH-deficient (7 cases). All KIT-mutant tumors were characterized by the presence of *KIT* mutation detected by Sanger sequencing. The SDH-deficient status was assessed by both immunohistochemistry of the SDHB subunit and Sanger sequencing of all the subunits of the SDH complex. Patients’ data are reported in Table 1, and additional details and clinical data are shown in Supplementary Table S1.

This study was approved by the institutional review board of IRCCS—Azienda Ospedaliero-Universitaria Policlinico S.Orsola-Malpighi, Bologna, Italy (approval number 113/2008/U/Tess). Each patient provided written informed consent.

Total RNA was extracted from tumor specimens with RNeasy Mini Kit (Qiagen, Milan, Italy) and then processed to be analyzed either on HGU133Plus 2.0 Affymetrix microarrays or by whole-transcriptome RNA sequencing on Illumina platform.

Briefly, for microarray samples, quality-controlled RNA was labelled following the Affymetrix manufacturer’s recommendations and then hybridized to HGU133Plus 2.0 arrays. Gene expression data were normalized and quantified as log2signal by the robust multichip average (RMA) algorithm (package oligo, R-bioconductor).

For the whole-transcriptome samples, the cDNA libraries were synthesized starting from 250 ng total RNA with TruSeq RNA Exome (Illumina, San Diego, CA, USA) according to the manufacturer’s protocol. Sequencing by synthesis was performed on Nextseq500 sequencer (Illumina) at 75 bp in paired-end mode. An average of 49.5 million reads per sample were obtained, reaching an average coverage of ~45×. Read pairs were mapped on reference human genome hg38 with STAR (https://github.com/alexdobin/STAR accessed 15 October 2020), duplicates removed, and sorting and indexing were performed with samtools (http://www.htslib.org/ accessed 15 October 2020). Gene expression was quantified and normalized in two different ways: (1) as count per million (CPM) by adopting the python package HTseq-count to get the raw count (https://htseq.readthedocs.io/ accessed 15 October 2020), followed by the R-bioconductor package edgeR to compute the normalization factors (https://bioconductor.org/packages/release/bioc/html/edgeR.html accessed 15 October 2020); (2) as transcript per million (TPM) using the program kallisto (https://pachterlab.github.io/kallisto accessed 15 October 2020). The two normalization methods are conceptually different and suited to perform different types of downstream analysis; generally CPM are employed to compare between samples while TPM are best suited to compare between genes [9]. Here, we adopted CPM to perform the principal component analysis (PCA) and the evaluation of differential expression (DE), and the TPM values were considered to estimate the tumor microenvironment composition and to quantify the gene signatures.

The R package prcomp (https://cran.r-project.org/package=nsprcomp accessed 15 October 2020) was adopted to perform the PCA, and the three-dimensional projections, corresponding to the first three components, were plotted with the function plot3d of rgl package (https://cran.r-project.org/package=rgl accessed 15 October 2020). The DE analysis of SDH-deficient versus KIT-mutant GISTs was conducted using the R-bioconductor limma package (https://www.bioconductor.org/packages/release/bioc/html/limma.html accessed 15 October 2020), sequentially adopting the functions lmFit (to produce a fitted model) and eBayes (to compute moderate t-statistic and log2 fold change). Significantly modulated genes (over- or underexpressed) were defined on the basis of *q*-value < 0.05 (adjustment method Benjamini–Hochberg). The methods described for PCA and DE was applied to both microarray and RNA-seq data series. Over-representation analysis was performed separately for over- and underexpressed gene lists to determine whether genes associated to a specific pathway are present more than expected. We adopted the web tool Enrich (https://maayanlab.cloud/Enrichr/ accessed 15 October 2020) focusing on MSigDB Hallmark 2020 to evaluate pathways and Human Gene Atlas to evaluate cell type. As input, we entered the two lists of up- and down-regulated genes obtained by the consensus between microarray or RNA-seq DE results. We included significantly modulated genes (*q*-value < 0.05) in microarray data having the same fold change sign and *p*-value < 0.01 in RNA-seq analysis (and vice versa). We also performed gene set enrichment analysis (https://www.gsea-msigdb.org/gsea/index.jsp accessed 15 October 2020) adopting the full expression matrix (without any filter) for both microarray and RNA-seq data. We ran Gene Set Enrichment Analysis (GSEA) by selecting the curated gene set carrying “canonical pathways” from Molecular Signatures Database (MSigDB) and adopting the following parameters: number of permutation = 100; enrichment statistic = “classic”; metric for ranking gene = “Diff_of_Classes”; normalization mode = “meandiv”. A tumor microenvironment study was performed using CIBERSORT, and immuno-related gene signatures were evaluated as previously described [10].

## 3. Results

### 3.1. Gene Expression Profile of SDH-Deficient GIST

Gene expression analysis was performed separately for fresh frozen tissue samples analyzed with microarray and FFPE samples analyzed with RNA-seq.

**Figure 1:**
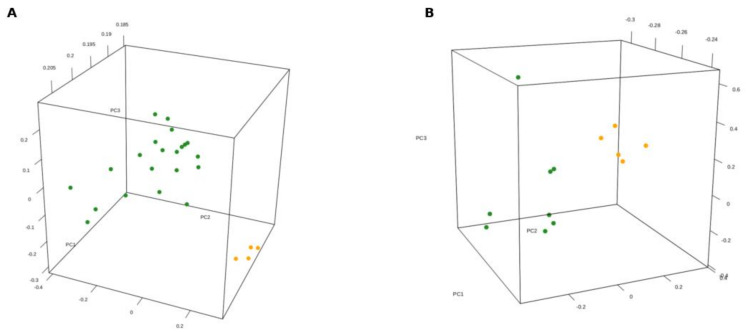
Principal component analysis (PCA) performed on samples analyzed with microarray ((**A**) number of SDH-deficient GISTs = 4; number of KIT-mutant GISTs = 21) and RNA-seq ((**B**) number SDH-deficient GISTs = 5; number of KIT-mutant GISTs = 8). The unsupervised PCA analysis shows the separation along the second component between the KIT-mutant groups (green points) and the SDH-deficient GISTs (orange points).

As first step, PCA was adopted to perform an unsupervised analysis with the aim to decompose the high dimensionality of transcriptome data variability into three-dimensional components. The 3D projections in both PCA analyses showed that SDH-deficient GISTs distinctly separate from KIT-mutant GISTs, providing proof of an expression profile typical of this molecular subgroup and profoundly different from KIT-mutant GISTs, supporting the hypothesis that the two GIST molecular groups may derive from two distinct cell types or oncogenic programs (Figure 1A,B).

The analysis of DE was performed for both sample series to discover the set of genes that are significantly overexpressed or down-regulated in SDH-deficient GISTs. For fresh frozen samples, analyzed by microarray, we found 833 and 928 genes that were respectively up- and down-regulated (adjusted *p*-value < 0.05); for the FFPE samples, analyzed by RNA-seq, 577 genes were overexpressed and 889 genes were underexpressed (adjusted *p*-value < 0.05) (Supplementary Tables S2 and S3).

Then, the data were intersected to identify the over/underexpressed genes commonly modulated in the two series of samples, highlighting 405 overexpressed and 331 underexpressed genes in SDH-deficient GISTs (Supplementary Table S4). These two sets of genes were adopted to perform the over-representation analysis with the Enrich web tool, as described in the method section. The significantly over-represented pathways (adjusted *p*-value < 0.05) for up- and downregulated genes are reported in Table 2. Among the upregulated pathways in the SDH-deficient group, we found hedgehog signaling, hypoxia, glycolysis, and epithelial-to-mesenchymal transition (EMT). Conversely, the set of underexpressed genes returned terms related to immune system, such as interferon gamma/alpha response, IL/STAT signaling, TNF-alpha signaling, and complement; moreover, fat metabolism and KRAS signaling were also highlighted. Interestingly, also the cell type over-representation analysis of down-regulated genes showed significant terms related to hematopoietic lineage, such as CD33+ myeloid, CD14+ monocytes, and CD56+ natural killer (NK) Cells; while the list of up-regulated genes produced a set of over-represented cell type mainly imputable to neuronal and brain tissues such as Fetal brain, pineal gland, prefrontal cortex, and superior cervical ganglion (Table 2, and complete results in Supplementary Table S5).

To further investigate the presence of enriched and depleted pathways in SDH-deficient GISTs, the whole expression matrices (form both microarray and RNA-seq analysis) were adopted to run the GSEA tool. GSEA offered a wide and complex picture of gene expression profile in our GIST series, and the full results are reported in Supplementary Tables S6 and S7. In order to highlight the strongest and clearest signals, we decided to intersect the results given by RNA-seq and microarray data. We found three shared significant pathways in SDH-deficient GISTs corresponding to the fibroblast growth factor receptor (FGFR) signaling, the glycosamminoglicane (GAG) degradation, including a group of lysosomal enzymes involved in the GAGs breakdown, and VXPX CARGO TARGETING TO CILIUM (the process of driving membrane proteins containing the motif valine-X-proline-X in the C-terminal tail towards the ciliary membrane) (Table 3). On the other hand, several commonly depleted pathways were highlighted by the GSEA analysis. Interestingly, we found immune system terms recurrence such as the cascade of Toll-like receptor complex, the IL3 pathway, the high-affinity IgE receptor signaling, and the granulocyte macrophage colony-stimulating factor.

Summing up, the gene set enrichment and the over-representation analysis showed a worthwhile scenario to be further explored. In particular, SDH-deficient GISTs appeared as a group of tumors with a presumed stand-alone histological background, marked by the activation of several gene signatures known to be related to invasion and tumor progression, and characterized by the depletion of immune competence. Given that, and based on the involvement in key oncogenic mechanisms, we decide to focus our investigation in neural-like signatures, FGFR signaling, hypoxia, EMT, and immune-related signatures.

### 3.2. Overexpression of Neural Markers

Among the top up-regulated elements in SDH-deficient GISTs, our analysis highlighted a relevant number of genes that are suggestive of neural commitment. Among them, we confirmed the overexpression of previously described (also validated by qPCR and IHC experiments) genes [11] such as the transcriptional regulator *LHX2* (known to be associated with the neural crest differentiation), the neurofilament light polypeptide *NEFL*, the synaptic cell adhesion molecule belonging to the nephrin-like family *KIRREL3*, the N-cadherin *CDH2*, and the neural progenitor-specific gene *IGF1R*. Moreover, the present analysis showed other important neural marker such as the glutamate receptor *GRIA1*, the integrin-α8 *ITGA8*, and the neuronal cell adhesion molecule *NRCAM*. This scenario, corroborating the hypothesis given by PCA analysis, suggested that SDH-deficient GISTs might derive from a diverse cell type (with respect to the more common GIST molecular subtype) and in particular, from cells committed to neural differentiation. We know that GISTs originate from mesenchymal cells, namely, interstitial cells of Cajal (ICC), located within the gastrointestinal tract and involved in the crosstalk between smooth muscle and nervous system. Recently, ICC were isolated from mice, and the transcriptome profile was deeply evaluated, identifying an important set of ICC markers [12]. Taking into account this set of genes (including *ANO1*, *KIT*, *PRCKCQ*, *THBS4*, *ELOVL6*, *GJA1*, *ADGRDA*, *EDN3*, *HPRT1*, and *ETV1*), we speculate if some difference exists in SDH-deficient GISTs with respect to KIT-mutant GISTs. We found that the majority of ICC markers were highly and equally expressed in both GIST subgroups; however, we found *THBS4* and *ELOVL6* that were more expressed, as well as *EDN3* and *GJA1* that were underexpressed in SDH-deficient GISTs (Supplementary Figure S1). The role of Endothelin-3 (EDN3) in the neural crest proliferation and differentiation was widely studied by Nagy at al. [13]. Interestingly, in this study, the presence of EDN3 in the hindgut explant cultures was clearly associated to the inhibition of neuronal differentiation. On the contrary, an increased expression of thrombospondin-4 (*THBS4*) was demonstrated to induce neuronal differentiation in CSPG4-expressing neural progenitor cells [14]. It is known that ICCs derive from mesenchymal stem cells that retain *KIT* expression during smooth muscle differentiation, probably due to induction from the nearby neural crest cells in the primitive gut that will give rise to the enteric nervous system [15,16]. ICCs are therefore cells that exhibit a relevant expression plasticity, which is probably reflected in their malignant counterparts. Taken together, all these connections validate the hypothesis that SDH-deficient GISTs could originate from a different type of ICC polarized towards a cell type with more pronounced neural features.

### 3.3. Fibroblast Growth Factor Receptor 2 Binding and Activation

**Figure 2:**
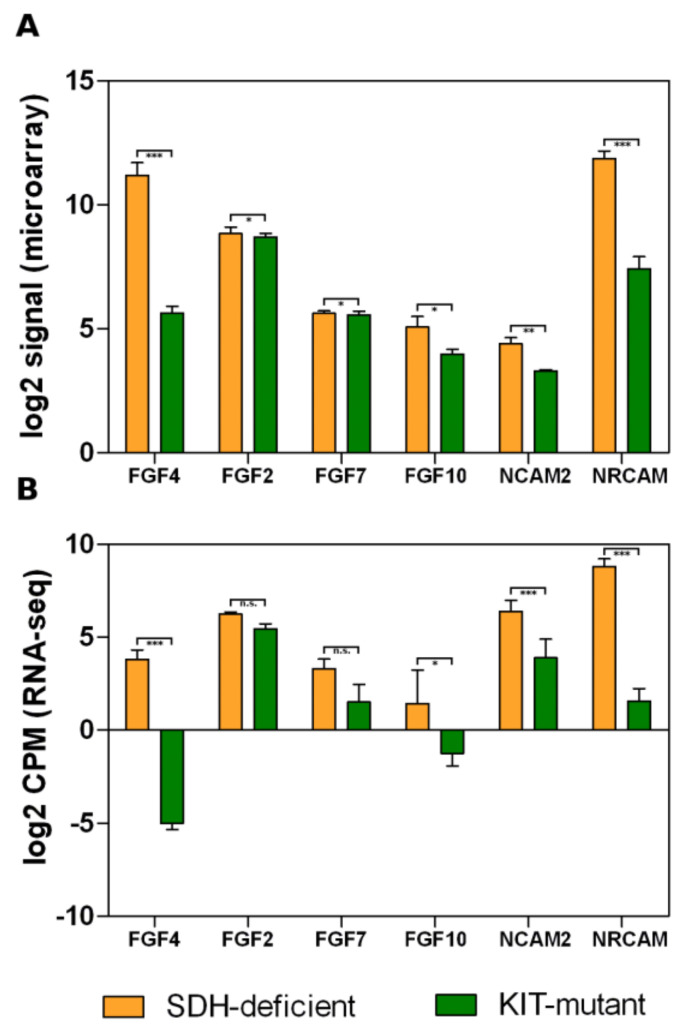
Box plots showing differences in expression levels of FGFs and NCAMs for the microarray series (**A**) and RNA-seq series (**B**). Orange boxes represent SDH-deficient GISTs; green boxes represent KIT-mutant GISTs. Significance level: *** *p*-value < 0.001; ** 0.001 ≤ *p*-value < 0.01; * 0.01 ≤ *p*-value < 0.05; n.s., not significant.

The pathway enrichment analysis showed a significant up-regulation of signaling related to fibroblast growth factors (FGFs) activation and the corresponding receptors (FGFRs) cascade. Notably, no FGFR genes were differentially modulated in SDH-deficient with respect to KIT-mutant GISTs. However, *FGFR1* and *FGFR2* showed a high expression level in all samples, while *FGFR3* and *FGFR4* abundance was close to zero. In contrast, we found a relatively large set of FGF ligands that are significantly highly expressed in the SDH-deficient group, such as *FGF4*, *FGF2*, *FGF7*, and *FGF10* (Figure 2). Interestingly, also the cell adhesion molecules *NRCAM and NCAM2* were strongly up-regulated in SDH-deficient GISTs. NCAMs family members are known to interact with FGFRs and to induce a specific FGFRs-mediated cellular response. In particular, the NCAMs–FGFRs interaction promotes the FGFRs stabilization and recycling to the cell surface of the receptors, indicating that FGFRs are activated by NCAMs in a very different way with respect to FGFs [17]. The concomitant presence of an increased level of both FGFs and NCAMs suggested that in SDH-deficient GISTs there are two different conditions possibly leading to FGFRs activation.

### 3.4. Comparison with SDH-Deficient Pheochromocytoma and Paraganglioma

Our analysis showed several interesting signatures overexpressed in SDH-deficient GISTs. To evaluate if the same expression profile and gene signatures were specifically characteristics of this rare subgroup of GIST, or if they were peculiar to neoplasms displaying the loss of function of SDH complex, we comparatively analyzed our microarray GIST series and a set of SDH-deficient pheochromocytoma and paraganglioma analyzed with the same protocol Affymetrix HG-U133 Plus 2.0 (available at E-MTAB-733 ArrayExpress). This dataset was published by Loriot et al. [18] within a research paper in which they identified the EMT activation specifically associated with SDHB-mutant metastatic pheochromocytoma and paraganglioma, concluding that this process may be involved in the acquisition of the invasiveness.

**Figure 3:**
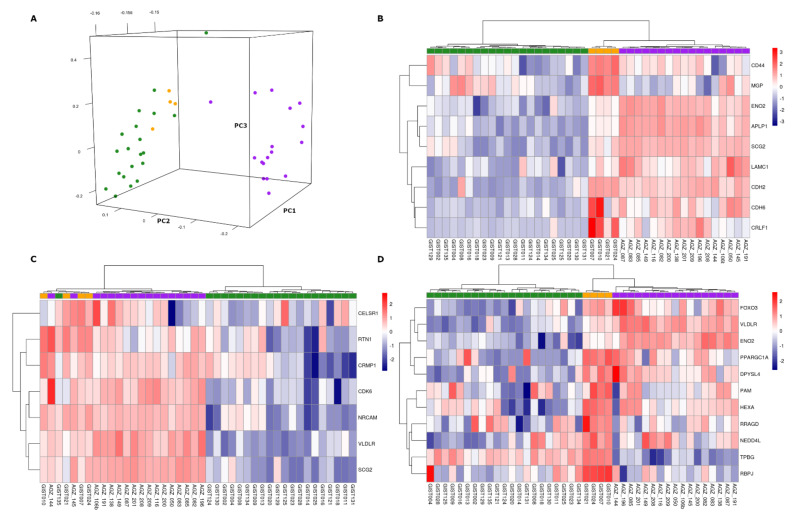
Comparative analysis between GISTs analyzed with microarray (number of SDH-deficient GISTs = 4; number of KIT-mutant GISTs = 21) and pheochromocytoma/paraganglioma carrying succinate dehydrogenase complex subunit B (SDHB) mutations. PCA highlights that GISTs (both SDH-deficient and KIT-mutant) cluster separately from pheochromocytoma/paraganglioma (**A**). Hierarchical clustering for the sets of genes emerged by enrichment analysis for the signaling of epithelial-to-mesenchymal transition (EMT) (**B**), hedgehog pathway (**C**), and hypoxia (**D**). Color labels orange, green, and purple correspond respectively to SDH-deficient GISTs, KIT-mutant GISTs, and pheochromocytoma/paraganglioma samples.

Firstly, we compared the whole expression profiles in an unsupervised manner, adopting PCA as previously described, putting together SDHB-mutant pheochromocytoma/paraganglioma and our microarray GIST samples (both SDH-deficient and KIT-mutant). The projections of the first three components show the GIST and pheochromocytoma/paraganglioma groups separately (Figure 3A). This result clearly suggests that the global gene expression specifically characterizes the two cancer types, probably due to the different histological derivation driving the transcriptional profile.

However, it is possible to hypothesize that specific signatures, which represent weaker signals with respect to the cell of origin, are due to the similarity of the genetic profile. So, we focused on the EMT pathway that, interestingly, also emerged as enriched in our SDH-deficient GISTs.

The acquisition of mesenchymal characters from epithelial cells, referred to as EMT, is normally associated to the embryonic development or to the tissue regeneration in adults. Moreover, EMT may occur during the tumor progression, inducing metastatic activity and increasing malignancy [19].

Several genes belonging to EMT signature are overexpressed in our SDH-deficient GISTs with respect to KIT-mutant GISTs. Among them, we found the cadherins *CDH2* and *CDH6*, the cytokine receptor *CRLF1*, the secretory protein *SCG2*, the amyloid precursor *APLP1*, the enolase *ENO2*, and the secreted protein *MGP*.

Taking into account this set of genes, a cluster analysis was performed. SDH-deficient GISTs and pheochromocytoma/paraganglioma were distinctly separate from KIT-mutant GISTs (Figure 3B), suggesting that the EMT expression pattern represents a shared feature in SDH-deficient tumors and is clearly different to KIT-mutant GISTs. In addition to the previously cited EMT genes, we also found the overexpression of the basic helix-loop-helix transcription factor *TWIST1* that is known to be associated to the EMT process and to play an important role in embryonic development, suggesting the existence of a diverse grade of differentiation shifted towards an early stage.

Notably, SDH-deficient GISTs showed the up-regulation of hedgehog signaling, another pathway strongly related to cell differentiation and cancer invasion. Similarly to the EMT pathway, we found that hedgehog signaling genes produced clusters that separate KIT-mutant GISTs and SDH-deficient (GISTs and pheochromocytoma/paraganglioma together), as shown in Figure 3C.

Finally, following the same procedure, we also performed the hierarchical clustering for hypoxia pathways. The cluster analysis showed that SDH-deficient GISTs and SDHB-mutant pheochromocytoma/paraganglioma shared the expression of hypoxia genes that is particularly evident for *FOXO3*, *VLDLR*, and *ENO2* (Figure 3D).

The hypoxia condition was associated with the overexpression of genes encoding for glutamate receptors [20], and in our data, as a matter of fact, we found the glutamate receptor *GRIA1* as one of the most up-regulated genes in SDH-deficient GISTs in both RNA-seq and microarray datasets.

### 3.5. SDH-Deficient GIST Immune Profiling

**Figure 4:**
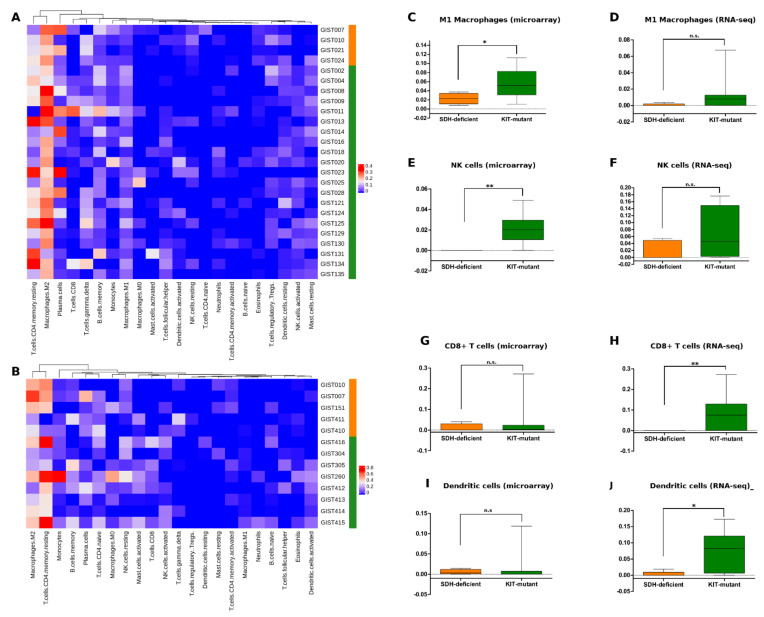
Tumor microenvironment analysis by CIBERSORT. Heatmaps represent the abundance of tumor-infiltrating cell subpopulations detected by microarray ((**A**) number of SDH-deficient GISTs = 4; number of KIT-mutant GISTs = 21) and RNA-seq ((**B**) number of SDH-deficient GISTs = 5; number of KIT-mutant GISTs = 8). Box plots (**C**–**J**) show the significant differences in the two GIST molecular subgroups of M1 macrophages, NK cells, CD8+ T-cells, and dendritic cells. Significance level is expressed as follow: ** *p*-value < 0.01; * 0.01 < *p*-value < 0.05; n.s., not significant. Orange labels correspond to SDH-deficient GISTs, while green labels correspond to KIT-mutant GISTs.

While the transcriptome profile of SDH-deficient GISTs has proved to be enriched in varied gene signatures supporting the histological origin, the oncogenic mechanism, and the tumor behavior, looking to the depleted signals, the leitmotiv appeared to be related to the immune landscape. Based on this observation, we applied CIBERSORT to comparatively evaluate the tumor microenvironment composition in the two GIST molecular subgroups. As well as for DE, CIBERSORT was run separately for microarray and RNA-seq data. The absolute and relative quantification of 22 hematopoietic populations is reported in the Supplementary Table S8; the absolute values were also adopted to build the heatmaps shown in Figure 4A,B. The analysis highlighted the M2 macrophages and the CD4+ T-cell memory resting as the more abundant cell types in both GIST groups. These observations are in agreement with what was previously described by several authors [10,21]. Overall, neither relative nor absolute abundance of tumor microenvironment subpopulations allowed to clusterize SDH-deficient GISTs separately from KIT-mutant GISTs; however, the t-test analysis at single subpopulation level depicted some noteworthy evidences. In particular, SDH-deficient GISTs in the microarray series showed a significantly lower abundance of M1 macrophages (*p*-value = 0.03) and NK cells (*p*-value < 0.01), and similar trends were found in RNA-seq data (Figure 4C–F). Moreover, SDH-deficient GISTs in RNA-seq samples showed a statistically significant lower level of CD8+ T-cells (*p*-value < 0.01) and dendritic cells (*p*-value = 0.03), which was also confirmed in the microarray series without reaching significance (Figure 4G–J). These results did not provide a definitely strong signal, probably due to the small and unbalanced sample number, however they unequivocally offered a picture that overlaps with the gene set enrichment and over-representation analysis described above, defining SDH-deficient GISTs as tumors with a cold tumor microenvironment.

**Figure 5:**
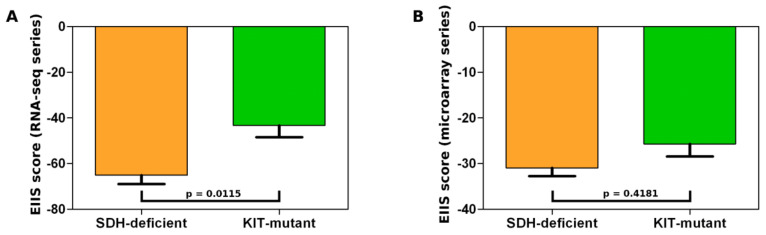
IFN-γ-induced immune signature (EIIS) score in SDH-deficient GISTs (orange box) and KIT-mutant GISTs (green box) computed for the microarray series (**A**) and for the RNA-seq samples (**B**).

We also evaluated specifically immune-related gene signatures previously analyzed in GIST [10] and first described as predictors of immunotherapy response [22,23], these are the expanded IFN-γ-induced immune signature (EIIS) and the T-cell-inflamed signature (TIS). We found that the EIIS score is lower in SDH-deficient GISTs (Figure 5). Even if we were not able to cluster SDH-deficient and KIT-mutant GISTs based on the EIIS signal of single genes (Supplementary Figures S2 and S3), we can observe a lower average EIIS score in SDH-deficient GISTs (Figure 5A,B), likely driven by few EIIS genes (including *CXCL10*, *STAT1*, and *HLA-E*) that are significantly down-regulated in this GIST group.

**Figure 6:**
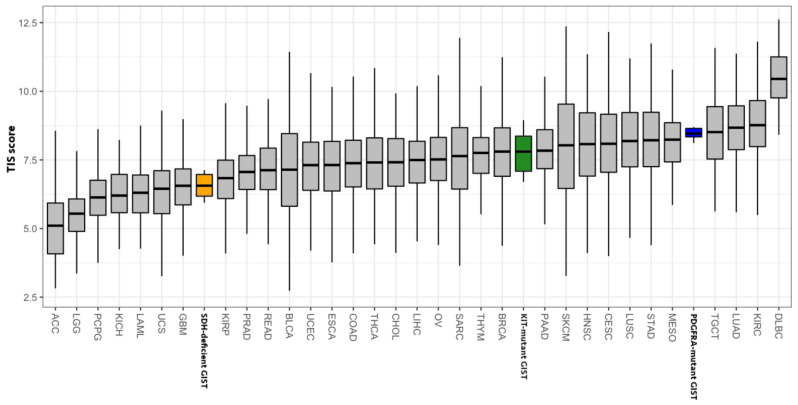
T-cell-inflamed signature score of GISTs (SDH-deficient GISTs in orange, KIT-mutant GISTs in green, and PDGFRA-mutant GISTs in blue) and other solid tumor types from The Cancer Genome Atlas (TCGA). ACC: adrenocortical carcinoma; BLCA: bladder urothelial carcinoma; BRCA: breast invasive carcinoma; CESC: cervical squamous cell carcinoma and endocervical adenocarcinoma; CHOL: cholangiocarcinoma; COAD: colon adenocarcinoma; DLBC: lymphoid neoplasm diffuse large B-cell lymphoma; ESCA: esophageal carcinoma, GBM: glioblastoma multiforme; HNSC: head and neck squamous cell carcinoma; KICH: kidney chromophobe; KIRC: kidney renal clear cell carcinoma; KIRP: kidney renal papillary cell carcinoma; LAML: acute myeloid leukemia; LGG: brain lower-grade glioma; LIHC: liver hepatocellular carcinoma; LUAD: lung adenocarcinoma; LUSC: lung squamous cell carcinoma, MESO: mesothelioma; OV: ovarian serous cystadenocarcinoma; PAAD: pancreatic adenocarcinoma; PCPG: pheochromocytoma and paraganglioma; PRAD: prostate adenocarcinoma; READ: rectum adenocarcinoma; SARC: sarcoma; SKCM: skin cutaneous melanoma; STAD: stomach adenocarcinoma; TGCT: testicular germ cell tumors; THCA: thyroid carcinoma; THYM: thymoma; UCEC: uterine corpus endometrial carcinoma; UCS: uterine carcinosarcoma; UVM: uveal melanoma.

Following the same procedure adopted by our group [10], we also evaluated the TIS score in our GIST series, comparing the results with the TIS score distribution in tumor types collected in The Cancer Genome Atlas (TCGA) database. Interestingly, we found that SDH-deficient GISTs showed TIS scores closer to glioblastoma multiforme and kidney renal papillary cell carcinoma, while KIT-mutant GISTs placed near to breast cancer and pancreatic adenocarcinoma. For sake of clarity, we included in this analysis also the GISTs of our previous series [10], excluding the KIT-mutant GISTs and leaving the PDGFRA-mutant GISTs. In strong agreement with our previous findings [24], PDGFRA-mutant GISTs are confirmed as the most immunogenic GIST molecular subgroup, showing a TIS score very similar to that of tumor types known to benefit from immunotherapy (such as lung cancer) (Figure 6). On the contrary, the TIS data obtained for SDH-deficient GISTs, paired to a lower EIIS expression and to the tumor immune microenvironment depletion, suggested that this GIST subgroup should be considered a noninflamed tumor for which immunotherapeutic approaches are far from being taken into consideration.

## 4. Discussion

In this study, we compared the gene expression profile between two different molecular groups of GIST, SDH-deficient and KIT-mutant, using a retrospective collection of RNA-seq and microarray data.

We identified distinct transcriptional profiles of SDH-deficient with respect to KIT-mutant GISTs, confirming what was previously described [25]. Moreover, we found interesting signaling pathways in SDH-deficient GISTs that may lead to useful information related to pathogenesis and to potential therapeutic targets.

Differential expression analysis, followed by over-representation and gene set enrichment analysis, revealed among the up-regulated pathways in the SDH-deficient group those of FGFR signaling, hypoxia, and EMT. Moreover, the SDH-deficient group showed a gene signature mainly characterized by overexpression of neural markers. Conversely, among the underexpressed pathways there are the interferon gamma/alpha response, KRAS and mTORC1 signaling, and fatty acid metabolism and complement. Interestingly, the immune-related signatures seem to be under-represented with respect to other GIST subgroups such as the PDGFRA-mutant GIST [21,24].

Our data confirmed the expression of markers related to neural development. As previously described, we found a high expression of genes *LHX2*, *NEFL*, *KIRREL3*, *CDH2*, and *IGF1R*. Furthermore, the present analysis showed other important neural markers such as the glutamate receptor *GRIA1*, the integrin-α8 *ITGA8*, and the neuronal cell adhesion molecule *NRCAM*. These results lead us to assume that the SDH-deficient GIST group originates from cells committed to the neural lineage. Recently, Young et al. have deeply evaluated the ICC transcriptome profile identifying an important set of ICC markers [11,12]. Considering the hypothesis of a different molecular origin, we further investigated by analyzing the expression of ICC markers in the two groups of samples. We realized that the majority of ICC markers were highly and equally expressed in both GIST subgroups; however, we found a modulated expression of some ICC markers know to be involved in the neuronal differentiation, such as *THBS4* and *EDN3* [13,14].

In our series, two members of the transmembrane receptor tyrosine kinases family, *FGFR1* and *FGFR2*, were highly expressed in all samples, while *FGFR3* and *FGFR4* showed a lower level of expression. Conversely, we found a differential expression profile in two groups for FGF ligands. In particular, *FGF4*, *FGF2*, *FGF7*, and *FGF10* showed a significantly high expression in the SDH-deficient group.

The cell surface receptor FGFR2 belongs to the human immunoglobulin superfamily. Its tyrosine kinase activity, triggered by extracellular ligand interaction and subsequent autophosphorylation, is involved in relevant biological processes including cell differentiation and mitogenesis, migration, and apoptosis. The extracellular portion of the receptor, carrying the Ig domains, may interact with the secreted FGFs or with other membrane proteins, including the neural cell adhesion molecules, NCAMs (also up-regulated in our SDH-deficient GIST series).

A large number of studies indicated that the deregulation of FGF signaling leads to many types of cancer (including hematological malignancies, breast cancer, and sarcomas), in which the genetic driver could be FGFRs translocations, amplifications, point mutations leading to FGFRs activation, or an increased level of autocrine or paracrine ligand stimulation [26].

The involvement of FGF/FGFR signaling to GIST pathogenesis was established in different molecular subgroups. It has been shown that in SDH-deficient GISTs, methylation of an FGF insulator region is responsible for the induction of *FGF4* expression [27,28]. *FGF3–FGF4* locus topology is profoundly altered in SDH-deficient GISTs, with *CTCF* insulator loss allowing aberrant expression of FGFR ligand genes [29]. We also recently confirmed that overexpression of the *FGF4* oncogene is related to an epigenetic status of FGF4 in GIST [30].

Interestingly, the up-regulation of genes encoding for the lysosomal enzymes, such as *SGSH*, *HGSNAT*, *HEXA*, *HEXB*, *NAGLU*, and *ARSB*, acting in the degradation of the main GAG groups, was reported in the SDH-deficient GIST group. GAGs are a family of complex polysaccharides known to play a crucial role in the cell biology, interacting with different growth factors and other transient components of the extra cellular matrix [31]. These molecules have been widely reported as modulators of the tumorigenic process by controlling signaling loops leading to unregulated cell growth, cancer progression, angiogenesis, and metastasis [32].Particularly interesting is the fact that specific groups of GAGs, such as the heparan sulfates, are able to trigger cell proliferation mechanisms through fibroblast growth factors (FGF1 and FGF2), vascular endothelial growth factor (VEGF), and transforming growth factor-β signaling [32,33].

Furthermore, we compared our GIST series with a set of SDH-deficient pheochromocytoma/paraganglioma in order to understand if the molecular signature of SDH-deficient GISTs was peculiar of GIST or related to the loss of the succinate dehydrogenase complex.

In the SDH-deficient GIST series, several genes belonging to EMT signature are overexpressed. Among them, we may list the cadherins *CDH2* and *CDH6*, the cytokine receptor *CRLF1*, the secretory protein *SCG2*, the amyloid precursor *APLP1*, the enolase *ENO2*, and the secreted protein *MGP*.

Loriot et al. performed transcriptional profiling to better understand the participation of EMT in the metastatic evolution of pheochromocytoma/paraganglioma. They identified the pathways that distinguishes SDHB-metastatic from all other types of pheochromocytoma/paraganglioma and suggest that activation of the EMT process might be associated to the particularly invasive phenotype of this group of tumors [18]. Our cluster analysis shows a separation of SDH-deficient GISTs together with pheochromocytoma/paraganglioma from KIT-mutant GISTs, suggesting that this pattern is common to tumors sharing a deficiency of the succinate dehydrogenase complex.

Several studies had previously supported that EMT features can be affected by genetic aberrations in the Krebs cycle enzymes, proving that the metabolic rewiring could be linked to cell plasticity and oncogenic transformation [34]. In particular, the inhibition of expression of SDH genes was associated with EMT activation in breast cancer [35], showing that the SDH loss-of-function can be a causative factor for EMT in tumors. Actually, many authors have described some type of sarcomas (such as synovial sarcomas, Ewing sarcoma, and uterine carcinosarcomas) as presenting an intermediate behavior between mesenchymal and epithelial stages named “metastable” phenotype [36]. This scenario is supported in our SDH-deficient series by the up-regulation of EMT marker N-cadherin *CDH2*, moreover, the expression of specific markers, like *TWIST1*, corroborate the hypothesis that these tumors are blocked at an early stage of differentiation correlating with the not rare clinical evidence of metastatic presentation.

Similarly, SDH-deficient GISTs showed the up-regulation of hedgehog signaling involved in the regulation of cell differentiation and proliferation. Additionally, for the genes associated with this pathway we found evidences of similarity with SDHB-mutant pheochromocytoma/paraganglioma.

Lastly, we found the hypoxia signaling up-regulated in SDH-deficient GISTs. Similarly to the EMT and hedgehog pathways, we showed that genes related to hypoxia signaling produced clusters combining SDH-deficient GISTs with pheochromocytoma/paraganglioma and separating them from KIT-mutant GISTs. Hypoxia is a metabolic condition in which tissues show a low oxygen level leading to the failure to maintain cellular functions. Hypoxia is known to be directly implicated in the neoplastic transformation of cells, which change their pattern and characteristics in response to the microenvironment oxygen lacking. However, the hypoxia-induced phenotypes are observed in some tumors also in the absence of hypoxia; in these cases, it is referred to as pseudo-hypoxia. Several malignant features are associated with the hypoxic/pseudo-hypoxic condition, including stem cell-like trait, metabolic alterations, and EMT [37] as well as angiogenesis, invasion, metastasis [38]. This particular characteristic was widely described in the subgroup of pheocromocytoma and paraganglioma carrying mutations in *SDH* genes and *VHL*, often classified as a pseudo-hypoxic cluster. In these kinds of tumors, together with SDH-deficient GISTs, the dysregulation of tricarboxylic acid leads to the pseudohypoxia status [39], also promoting the anaerobic process of glycolysis [40] that, as matter of fact, is up-regulated in our SDH-deficient GIST series.

Taken together, these observations indicate that the expression of EMT, hedgehog, and hypoxia pathways is strongly linked to the SDH complex deficiency, a feature shared with pheochromocytoma/paraganglioma, which could explain and support clinically relevant differences with other GIST subgroups, such as for the metastatic behavior.

In this study, the immunological state of both groups of GISTs was evaluated. The results showed a significant absence of immune infiltrate in SDH-deficient patients, which indeed display a low abundance of tumor-infiltrating CD8+, M1 macrophages, NK cells, and dendritic cells. Moreover, the EIIS signature in SDH-deficient GISTs is lower than in KIT-mutant GISTs. By comparing the TIS score in our GIST series with the TIS score distribution in several tumor types (collected in TCGA database), we can see that the SDH-deficient TIS score is closer to that of glioblastoma multiforme and kidney renal papillary cell carcinoma, emerging as the lowest with respect to other GIST molecular subgroups (both KIT-mutant and PDGFRA-mutant) that showed a TIS score more similar to hot tumors (such as melanoma and lung cancer).

These findings lead us to assume that SDH-deficient GISTs are noninflamed cancers with a poor tumor microenvironment and definitely different from other GIST groups for which several studies have speculated about the possible efficacy of immunotherapeutic approaches [10,21,41].

## 5. Conclusions

This study delves into the expression landscape of SDH-deficient GISTs and highlights gene expression pattern similarity and differences with respect to the most common KIT-mutant GISTs and with respect to other neoplasm carrying an analogous molecular background leading to the SDH loss of function. These findings could help the scientific community of oncologists, pathologists, and biologists to better understand both the histology and the dysregulated biological processes as putative target of new therapeutic strategies.

## Figures and Tables

**Table 1:** Summary of characteristics of patients with succinate dehydrogenase deficiency (SDH-deficient) and proto-oncogene c-Kit mutant (KIT-mutant) gastrointestinal stromal tumors (GISTs).

	SDH-Deficient	KIT-Mutant
**Sex**		
Female	4	11
Male	3	18
**Age (average, range)**	25 (18–30)	62.4 (34–87)
**Site**		
Intestine	0	9
Stomach	6	17
NA	1	3
**Tumor size**		
<5 cm	0	4
≥5 cm	5	22
NA	2	3
**Mitotic rate**		
<5/50 HPF *	0	9
≥5/50 HPF	4	15
NA	3	5
**Desease status at diagnosis**		
Localized	0	18
Metastatic	5	7
NA	2	4
**Platform**		
Microarray	4 **	21
RNA-seq	5 **	8

* High power fields. ** GIST010 and GIST007 with both platforms.

**Table 2:** Over-representation analysis by Enrich tool.

Database	Terms	*p*-Value
MSigDB Hallmark up-regulated genes	Hedgehog Signaling	0.00001
	Hypoxia	0.00263
	UV Response Dn	0.0027
	Estrogen Response Early	0.00779
	Glycolysis	0.00779
	Apical Junction	0.02104
	Epithelial Mesenchymal Transition	0.02104
MSigDB Hallmark down-regulated genes	TNF-alpha Signaling via NF-kB	0.00001
	Interferon Gamma Response	0.00013
	KRAS Signaling Up	0.00013
	Fatty Acid Metabolism	0.00031
	IL-2/STAT5 Signaling	0.0005
	Complement	0.0019
	IL-6/JAK/STAT3 Signaling	0.00322
	Bile Acid Metabolism	0.01086
	Adipogenesis	0.01857
	Interferon Alpha Response	0.02256
Human Gene Atlas up-regulated genes	Fetalbrain	0
	pineal night	0.00001
	PrefrontalCortex	0.00043
	Amygdala	0.00072
	pineal day	0.00412
	SuperiorCervicalGanglion	0.01149
	Thyroid	0.01317
	CerebellumPeduncles	0.02167
	BronchialEpithelialCells	0.02805
Human Gene Atlas down-regulated genes	CD33+ Myeloid	0.00018
	CD14+ Monocytes	0.00069
	CD56+ NKCells	0.00173
	Prostate	0.00303
	Liver	0.0151
	BronchialEpithelialCells	0.01868
	Adipocyte	0.03136

**Table 3:** Significantly enriched pathways highlighted in both microarray and RNA-seq series by Gene Set Enrichment analysis (GSEA).

	Microarray		RNA-seq	
Pathway NAME	NES *	*p*-Value	NES	*p*-Value
REACTOME PHOSPHOLIPASE C MEDIATED CASCADE FGFR2	1.802	0.022222	1.354	0
REACTOME_FGFR2_LIGAND_BINDING_AND_ACTIVATIONC **	1.717	0.044444	1.313	0.0625
WP GLYCOSAMINOGLYCAN DEGRADATION	1.686	0.041667	1.346	0.02564
REACTOME VXPX CARGO TARGETING TO CILIUM	1.576	0.019231	1.276	0.040816

* Normalized enrichment score. ** REACTOME_FGFR2_LIGAND_BINDING_AND_ACTIVATION showed a *p*-value of 0.0625 in the RNA-seq data that is slightly lower with respect to the significance threshold adopted.

## Data Availability

The data are available on request by contacting Valentina Indio (valentina.indio2@unibo.it) or the corresponding author.

## Supplementary Materials

The following are available online at https://www.mdpi.com/2077-0383/10/5/1057/s1: Supplementary Tables S1: Patient’s characteristics, Supplementary Table S2: Significantly modulated genes detected by microarray technology, Supplementary S3: Significantly modulated genes detected by RNA-seq technology, Supplementary Table S4: Commonly down- and up-regulated genes, Supplementary Table S5: Over-representation analysis performed with Enrich web tool, Supplementary Table S6: List of significantly depleted pathways in SDH-deficient detected with Gene Set Enrichment Analysis (GSEA), Supplementary Table S7: List of significantly enriched pathways in SDH-deficient detected with GSEA, Supplementary Table S8: Relative and absolute quantification of 22 hematopoietic microenvironment cell sub-populations computed by CIBERSORT; Supplementary Figures S1:Box plots showing differences of expression level of neural markers in RNA-seq, Supplementary Figure S2: Heatmap representing the level of expression of IFN-γ-induced immune signature (EIIS) in SDH-deficient GIST (orange) and KIT-mutant (green) in microarray samples, Supplementary Figure S3: Heatmap representing the level of expression of EIIS in SDH-deficient GIST (orange) and KIT-mutant (green) in RNA-seq samples.
