# Supplementary material for: Gene Expression Landscape of SDH-Deficient Gastrointestinal Stromal Tumors
Source: J Clin Med. 2021 Mar 4;10(5):1057. doi: 10.3390/jcm10051057 (PMC7961685; doi:10.3390/jcm10051057)
Supplement: Supplementary file 1 [file jcm-10-01057-s001.zip › jcm-1112043 - Supplementary Figures.pdf]

Supplementary Figures

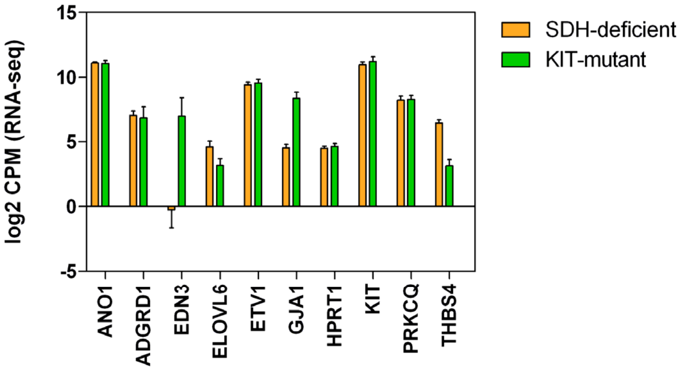

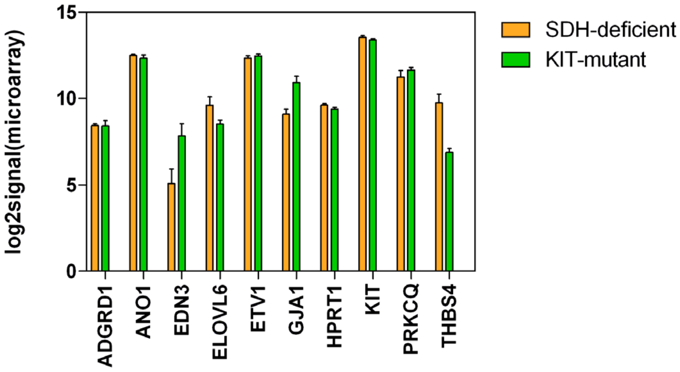

Supplementary Figure S1. Box plots showing differences of expression level of neural markers in RNA-seq (A) and microarray series (B). Orange boxes represent SDH-deficient GIST; green boxes represent KIT-mutant GIST.

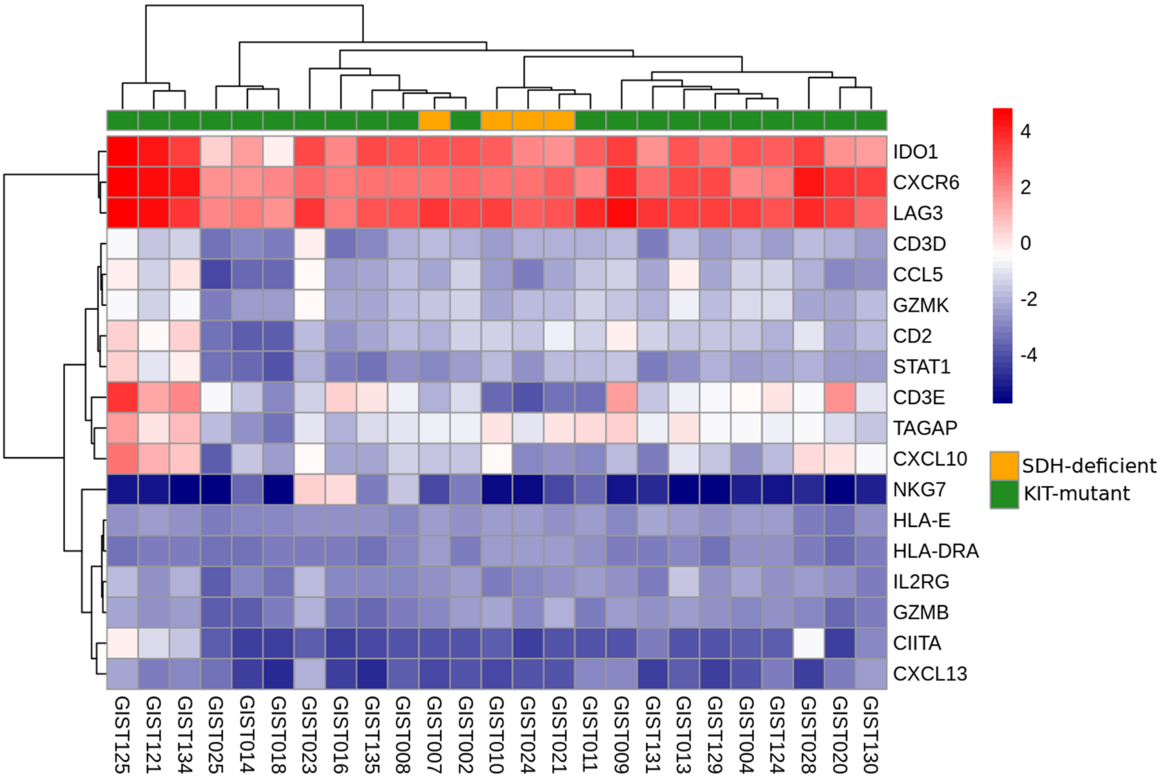

Supplementary Figure S2. Heatmap representing the level of expression of IFN-γ-induced immune signature (EIIS) in SDH-deficient GIST (orange) and KIT-mutant (green) in microarray samples.

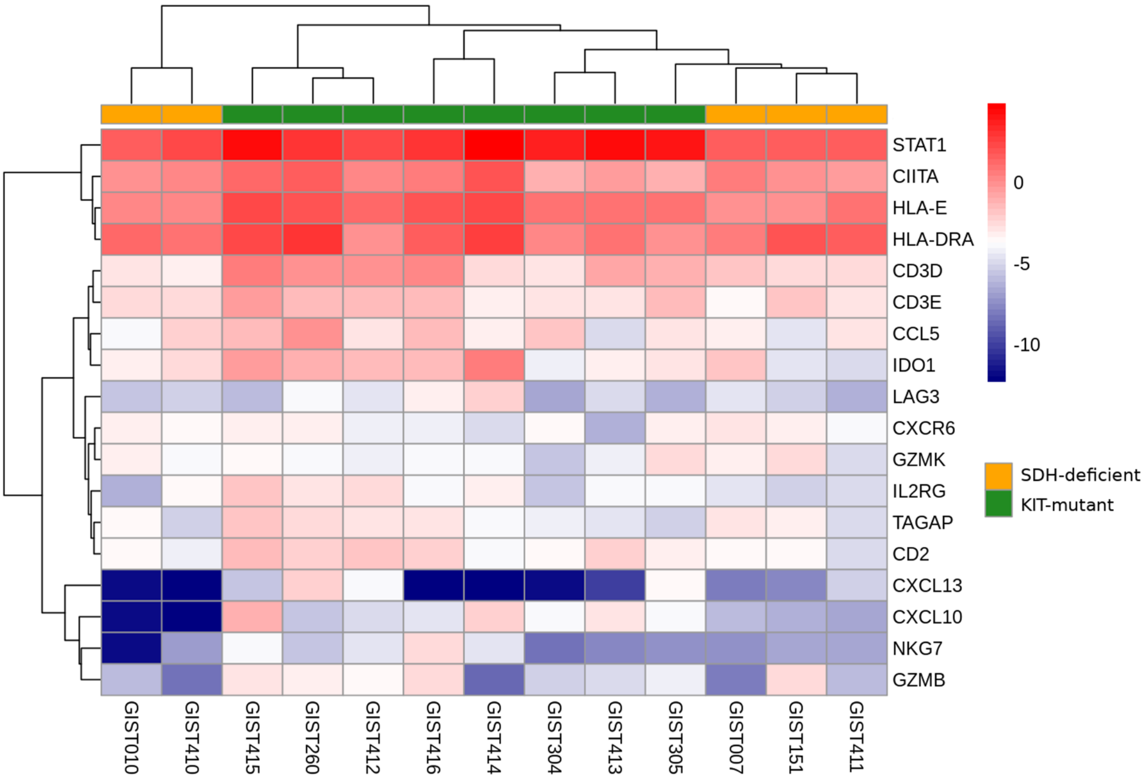

Supplementary Figure 3. Heatmap representing the level of expression of IFN-γ-induced immune signature (EIIS) in SDH-deficient GIST (orange) and KIT-mutant (green) in RNA-seq samples.
